# Oxidative, Genotoxic and Epigenotoxic Effects of *Pimpla turionellae* Venom at Pharmacological Perspective

**DOI:** 10.1007/s13744-025-01283-5

**Published:** 2025-05-22

**Authors:** Aslı Eskin, Zülbiye Demirtürk, Famil Yusufoğlu, Fevzi Uçkan

**Affiliations:** Department of Biology, Kocaeli University, İzmit, Kocaeli Türkiye

**Keywords:** *Galleria mellonella*, Parasitoid venom, Pharmaceutics, *Pimpla turionellae*

## Abstract

Insects and mammals share a similar innate immune system. *Galleria mellonella* (L.), a beekeeping pest, is an alternative model organism for human health studies due to its immune response similarity and ability to be maintained at 37 °C. While oxidative stress and genotoxicity cause diseases, antioxidant enzymes and epigenetic mechanisms are effective in immunological response processes. Although parasitoid venoms are potential candidates for pharmacological applications such as anticoagulant, antibiotic, painkiller, antiviral and anticancer agents, the information pool is scarce to reflect their effects in humans. In an attempt to reveal the pharmaceutical significance of parasitoid venoms and their potential effects on human health, different venom doses of *Pimpla turionellae* (L.), the solitary endoparasitoid of *G*. *mellonella*, were injected into the host. Then, the levels of protein content, advanced oxidised protein products, lipid peroxidation, antioxidant power and glutathione in host haemolymph, and the amounts of methylation marker 5-methyldeoxycytidine monophosphate and strand breakage rates under neutral and alkaline conditions in host DNA were analysed. Principal component analysis was performed to determine the number of components that oxidative parameters depend on, and multivariate correlation analysis was applied to evaluate the effects of the parameters on each other. It was concluded that *P*. *turionellae* venom appeared to be one of the most effective pharmaceutical agents among parasitoid venoms. Also, the 0.01 venom reservoir equivalent dose qualified as immunotherapeutic dose.

## Introduction

Insects are one of the most cosmopolitan animal taxa on our planet. They inhabit almost every habitat (Kavanagh and Reeves 2004). It is estimated that insects have around 750,000 species (Samways 1993), but recent studies reveal that this number is close to 1,000,000 (Zhang et al. 2011). Comprising about 66% of the species in the animal kingdom, insects are known to be one of the most diverse life forms (Ratcliffe 1985; Zhang et al. 2011). Although insects and vertebrates diverged from each other in evolutionary perspective about 500 million years ago, their physiology has remained similar in many aspects (Boman and Hultmark 1987). Although mammals have evolved an adaptive immune system apart from insects, the innate immune system of mammals and insects shows structural and functional homology and shares many common features (Arala-Chaves and Sequeira 2000; Fallon and Sun 2001; Hoffmann 1995; Klein 1997; Ratcliffe 1985; Salzet 2001).

The greater wax moth *Galleria mellonella* (L.) (Lepidoptera: Pyralidae) is one of the most severe pests of beekeeping due to the disruptive feeding behaviour of its larvae. They often live in beehives and feed on wax and pollen. They also cause devastation in beehives and lead to morbidity among bee and wasp species (Kwadha et al. 2017; Wojda 2017). They are preferred as reliable model organisms for human pathogen researches since they have cellular and humoral immune responses and can be maintained at 37 °C, which is the human body temperature (Cook and McArthur 2013; Smoot et al. 2001). *Pimpla turionellae* (L.) (Hymenoptera: Ichneumonidae), one of the species of parasitoids that are the hidden life vest of ecology, is a solitary, idiobiont endoparasitoid of numerous Lepidoptera species, including *G*. *mellonella* (Ergin et al. 2006; Kansu and Uğur 1984; Uçkan and Gülel 2002). *Pimpla* *turionellae* can suppress the host immune system with its venom constituents (Uçkan et al. 2004). Maternal factors such as the calyx fluid and venom content injected into the host by female wasps before laying eggs cause suppression of the host’s immune responses and thus parasitoid larvae can develop inside the host (Asgari and Rivers 2011; Beckage et al. 1994; Moreau and Asgari 2015).

Under normal physiological conditions, there is a balance between the reactive oxygen species and the antioxidant defence system that inactivates them (Halliwell and Whiteman 2004). Damage caused by reactive oxygen species that disrupt the structure of macromolecules in organisms is called oxidative stress. Oxidative stress negatively affects the immune system and causes many diseases (Aruoma and Halliwell 1998; Storz and Imlayt 1999). Any damage to the structure of genes, DNA and chromosomes is called genotoxicity. It can cause a wide range of problems and diseases, including cell death and cancer (Williams 1989). Epigenetics is the process of reversible and heritable alteration of gene expression by endogenous and environmental stimuli without changing the genetic structure (Glastad et al. 2019; Mukherjee and Dobrindt 2022; Palli 2021). There are six different epigenetic mechanisms: DNA modifications, histone modifications, histone variants, nuclear dynamics, chromatin remodelling and non-coding RNAs (Castro-Muñoz et al. 2023; Chen et al. 2021; Ferreira and Esteller 2018; Zhang et al. 2020). The most frequently investigated DNA modification is DNA methylation (Lande-Diner et al. 2007). During infection, the expression level of different protein classes involved in insect immune defence is regulated by epigenetic mechanisms (Gerardo et al. 2010; Heitmueller et al. 2017).

Due to convergent evolution, parasitoids are significantly miniaturised (Xu et al. 2021). In contrast, with divergent evolution, the content of parasitoid venoms has diverged depending on environmental factors and host-specificity (Cavigliasso et al. 2019; Yang et al. 2024). Because of their small size, low venom amounts and high host-dependence, it is very challenging to rear them on synthetic food in the laboratory and to collect enough samples to perform molecular analyses in replicates (Kamiyama et al. 2025; Shimada-Niwa et al. 2025; Yang et al. 2020). It is difficult to characterise parasitoid venoms by conventional methods because even parasitoids with a common hosts show variable venom contents (von Reumont et al. 2022; Yang et al. 2024). In addition, different approaches such as taxonomical classification, genomics, transcriptomics and proteomics are required to identify these tiny creatures and to determine their rich venom contents (Cavigliasso et al. 2021; Kamiyama et al. 2025; Poirié et al. 2014; von Reumont et al. 2022). By overcoming these problems with modern technologies and multidisciplinary approaches, the complex structure of parasitoid venoms can be elucidated. Also, their cross-species applicability can be improved by enabling their use in applied fields such as medicine and agriculture.

Serine proteases and enzymes with similar functions found in the venoms of parasitoids can inhibit blood clotting (Choo et al. 2010; Czaikoski et al. 2010; De Graaf et al. 2010; Furihata et al. 2013; Parkinson et al. 2002; Zhu et al. 2010). They may also harbour antimicrobial peptides in their venom or stimulate antimicrobial immune responses (Konno et al. 2007; Kuhn-Nentwig 2003; Moreau 2013; Shen et al. 2010; Zhu et al. 2011). The neurotoxic peptides in the venom can suppress the experience of pain by shutting down ion channels (Eldefrawi et al. 1988; Konno et al. 2001; Piek 1982). The venoms of several endoparasitoid species contain molecules that induce protease inhibition, apoptosis and cytotoxicity (Asgari 2012; Moreau and Guillot 2005). Due to such potential effects and their high stability, they are promising candidates for a wide range of pharmacological applications as anticoagulants in thrombotic diseases, antibiotics in microbial diseases, painkillers, antivirals and anticancer drugs (Moreau and Asgari 2015). On the other hand, although various biological effects of parasitoid venoms on hosts have been investigated, studies on the effects on human health are limited to a few accidental envenomation cases in the literature (Moreau 2013; Moreau and Asgari 2015). In prior studies with parasitoid venoms on *G*. *mellonella*, various effects were observed depending on the parasitoid species, the dose of the venom, the duration of exposure to the venom and the time elapsed after exposure to the venom (Aamer et al. 2024; Coates et al. 2019; Çim and Altuntaş 2021; Özbek et al. 2020). Therefore, the pharmacological status of parasitoid venoms and their doses should be elucidated. Here, the impacts of *P*. *turionellae* venom to host *G*. *mellonella* immunity were evaluated from oxidative, genotoxic and epigenetic points of view. Considering the immunological homology, the possible effects of parasitoid venoms in humans and their pharmaceutical significance were elucidated. Immunotherapeutic dose was determined according to the results of the analyses. As a conclusion, our findings may lead to the determination of the parasitoid with the highest potential pharmaceutical importance and its therapeutic dose for human health research.

## Material and Methods

### Animal Husbandry

Host and parasitoid insects were reared in the laboratory at 25 ± 3 °C and 60 ± 3% relative humidity conditions. *Galleria* *mellonella* was cultured in synthetic food consisting of a mixture of water, honey, bran, glycerin and honeycomb (Sak et al. 2006) kept in constant darkness. *Pimpla turionellae* was produced by oviposition on host pupae. They were cultured in a 12 h:12 h (L:D) photoperiod with pupae and honey supplementation for feeding.

### Derivation of Venom Doses and Treatment Groups

**Fig. 1:**
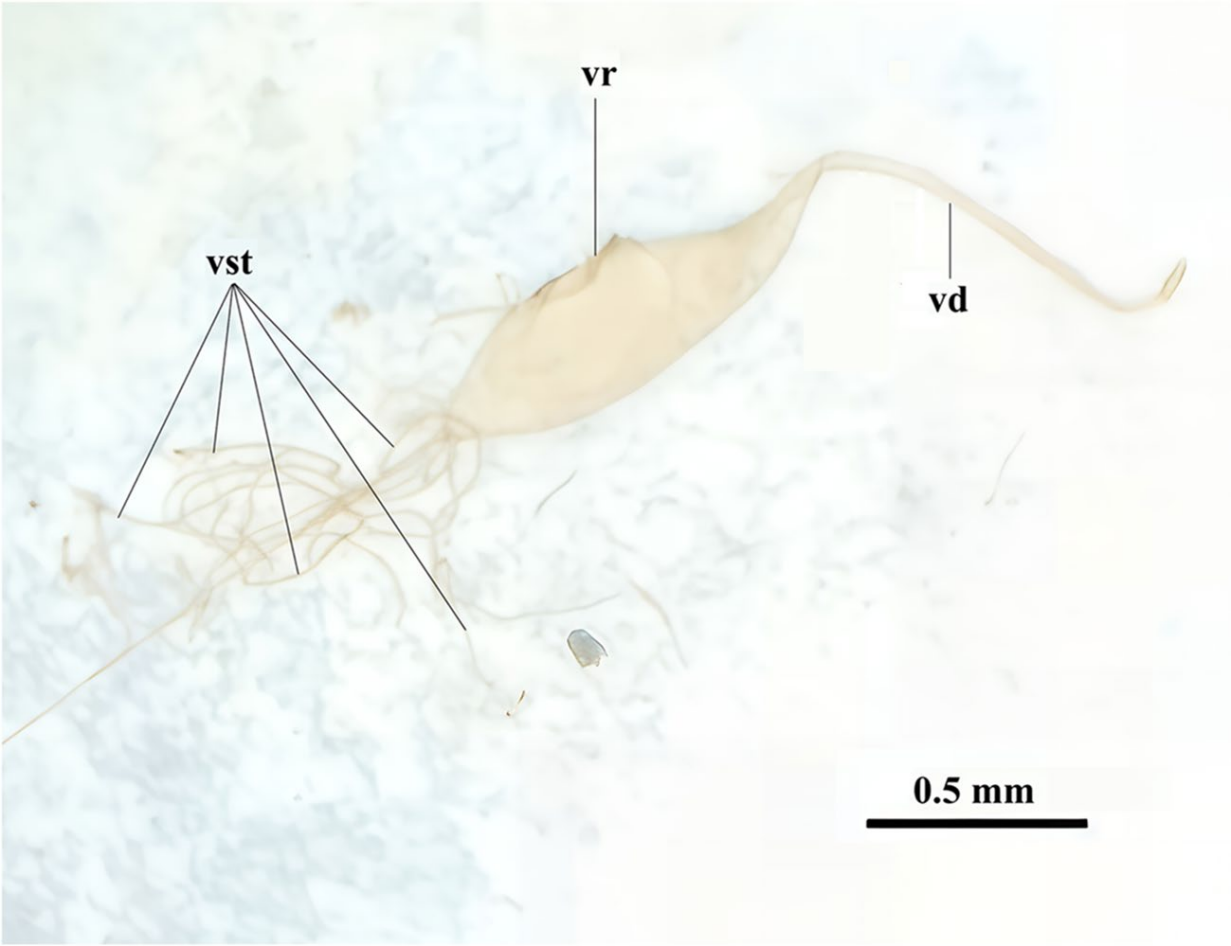
Morphological structure of extracted venom sac of *Pimpla turionellae*. vst, venom secretory tubules; vr, venom reservoir; vd, venom duct

The venom sacs of 15–20 days old non-parasitised *P*. *turionellae* females were pulled out from their ovipositors under a stereo microscope (Olympus SZ51) (Fig. 1). The venom contents of the sacs were transferred into tubes containing PBS. Five microliters of different venom doses, that was derived based on results of Ergin et al. (2006) probit analyses, was immediately injected into *G*. *mellonella* larvae using a syringe (Hamilton®) and the experimental groups were designed (Table 1). Following 24 h of waiting, haemolymph was collected from the larvae as 10 individuals from each group and treated with N-Phenylthiourea to prevent melanisation (Uçkan et al. 2004). To observe the inter-group changes in biochemical analyses in the most efficient way, haemolymph was diluted with PBS in accordance with spectrophotometric measurements. Samples at the appropriate concentration were stored at − 80 °C until used for analyses.

**Table 1:** Various doses of the venom of the parasitoid *Pimpla turionellae* administered on the host *Galleria mellonella*

Groups*	Venom amount	PBS amount
Control I	N/A	N/A
Control II	N/A	5 µL
0.005 VRE**	1 venom sac	1000 µL
0.01 VRE	1 venom sac	500 µL
0.05 VRE	1 venom sac	100 µL
0.1 VRE	2 venom sacs	100 µL
0.5 VRE	10 venom sacs	100 µL
1 VRE	20 venom sacs	100 µL

*For Control I group, a blank injection was applied, and for Control II group, 5 µL PBS was applied. For the remaining treatment groups, 5 µL of the indicated venom doses were applied

**VRE, venom reservoir equivalent

### DNA Isolation

Larvae were pulverised with liquid nitrogen via a porcelain mortar and pestle and DNAs were extracted as per the method of Chen et al. (2008). The amount and purity of the DNA isolates were measured by spectrophotometric method in BMG Labtech SPECTROstar^*Nano*^ device. DNA isolates were electrophoresed by agarose gel electrophoresis and visualised using Vilbert-Lourmat UV Transilluminator. DNA isolates of 10 larvae from each group with appropriate purity and quantity were stored for use in toxicity analyses.

### Biochemical Analyses

To quantify the oxidative effects of parasitoid venom, haemolymphs pre-collected were analysed using spectrophotometric techniques in triplicate. Total protein contents were determined by the bicinchoninic acid (BCA) method at 562 nm wavelength (Smith et al. 1985) and were used in the determination of other biochemical analyses. The levels of advanced oxidised protein products (AOPP) were determined by spectrophotometer at 340 nm and the results were evaluated with Chloramine T equivalence (Hanasand et al. 2012). Changes in the level of lipid peroxidation were observed by measuring malondialdehyde (MDA) levels at 532 nm (Buege and Aust 1978) and the results were calculated considering the extinction coefficient (ε = 1.56 × 10 − 5 M^−1^ cm^−1^). By Ferric Reducing Antioxidant Power (FRAP) assay, a method to quantify antioxidant power based on iron reduction reaction, the effect of parasitoid venom on host antioxidant system was determined by spectrophotometric measurements at 593 nm of haemolymph treated with FRAP reagent (300 mM acetate buffer, 10 mM 2,4,6-tripridyl-s-triazine, 20 mM FeCl_3_; 10:1:1) against ascorbic acid standards (Benzie and Strain 1996). The alteration in glutathione (GSH) levels among the groups was determined at 412 nm using dithionitrobenzoic acid (DTNB) (Beutler et al. 1963). The results of changes between groups were given as nmol/mg protein for AOPP and MDA assays and µmol/mg protein for FRAP and GSH analyses.

### Genotoxicity and Epigenotoxicity Analyses

To observe the genotoxic effects of the parasitoid venom, the DNA isolates extracted were analysed by simple electrophoretic assay for genomic DNA lesions quantification (EAsy-GeL) method (Londero et al. 2021). HyperLadder™ 1 kb (Bioline meridian BIOSCIENCE ®; Cat No: BIO-33026) was loaded in the first well of the agarose gel to measure DNA breakage rates. Results were given as DNA breaks per 1 base pair (bp). Double strand DNA breakage (DSB) and double and single strand DNA breakage (DSB + SSB) rates were calculated for neutral and alkaline gel electrophoresis, respectively.

Nucleotides were obtained from DNA isolates using DNA Degredase. Perkin Elmer P200 high-performance liquid chromatography (HPLC) was used to observe the effects of parasitoid venom on host DNA methylation levels. Samples were filtered in Corning Costar Spin-X eppendorf tubes and 20 µL of filtered samples was loaded onto an HPLC column (ACE 3 C8, 125 × 4.0 mm) equilibrated with ammonium orthophosphate buffer. Global DNA methylation levels were determined by calculating the area under the peaks of the samples for 5-methyldeoxycytidine monophosphate (5 m-dCMP). Using the area under the peaks of the 5 m-dCMP standard and the samples, the methylation rate of parasitoid venom on host DNA was calculated as 5 m-dCMP ng/1 µg DNA (Özbek et al. 2020; Ramsahoye 2002).

### Statistical Analyses

For each data acquired from biochemical and genetic analyses, Gaussian (normal) distribution analyses were performed with Levene’s test in IBM SPSS Statistics v27.0.1.0 software. For datasets that did not show normal distribution, the data were first normalised by taking the square root of the data. The Kruskal–Wallis analysis was performed for samples that were still not normally distributed. Post hoc analysis was carried out with Dunn’s test to observe statistically significant changes (*p* < 0.05). On the other hand, one-way analysis of variance (ANOVA) was applied to determine the homogeneity of the normally distributed data groups. Tukey’s Honestly Significant Difference (Tukey’s HSD) test was used as a post hoc test to evaluate whether there were statistically significant changes. In addition, principal component analysis (PCA) was performed in OriginLab OriginPro 2024 SR1 v.10.1.0.178 to determine the number of factors affecting the oxidative effect parameters. Finally, multivariate correlation analysis (MCA) based on Pearson’s method was performed to reveal the relationship between MDA, AOPP, DSB, DSB + SSB and DNA methylation levels induced by the venom on the host.

## Results

### Quantities and Purities of DNA

According to spectrophotometric measurements, DNA quantities were determined as approximately 5000 ng/µL for each larva. For the efficiency of the work, isolates with a purity level between 1.8 and 2.0 at the absorbance ratio A260/280 and between 2.0 and 2.2 at the absorbance ratio A260/230 and those that were well visualised in electrophoresis were selected (Lucena-Aguilar et al. 2016).

### Oxidative Effects of Venom and Factor Analysis

**Fig. 2:**
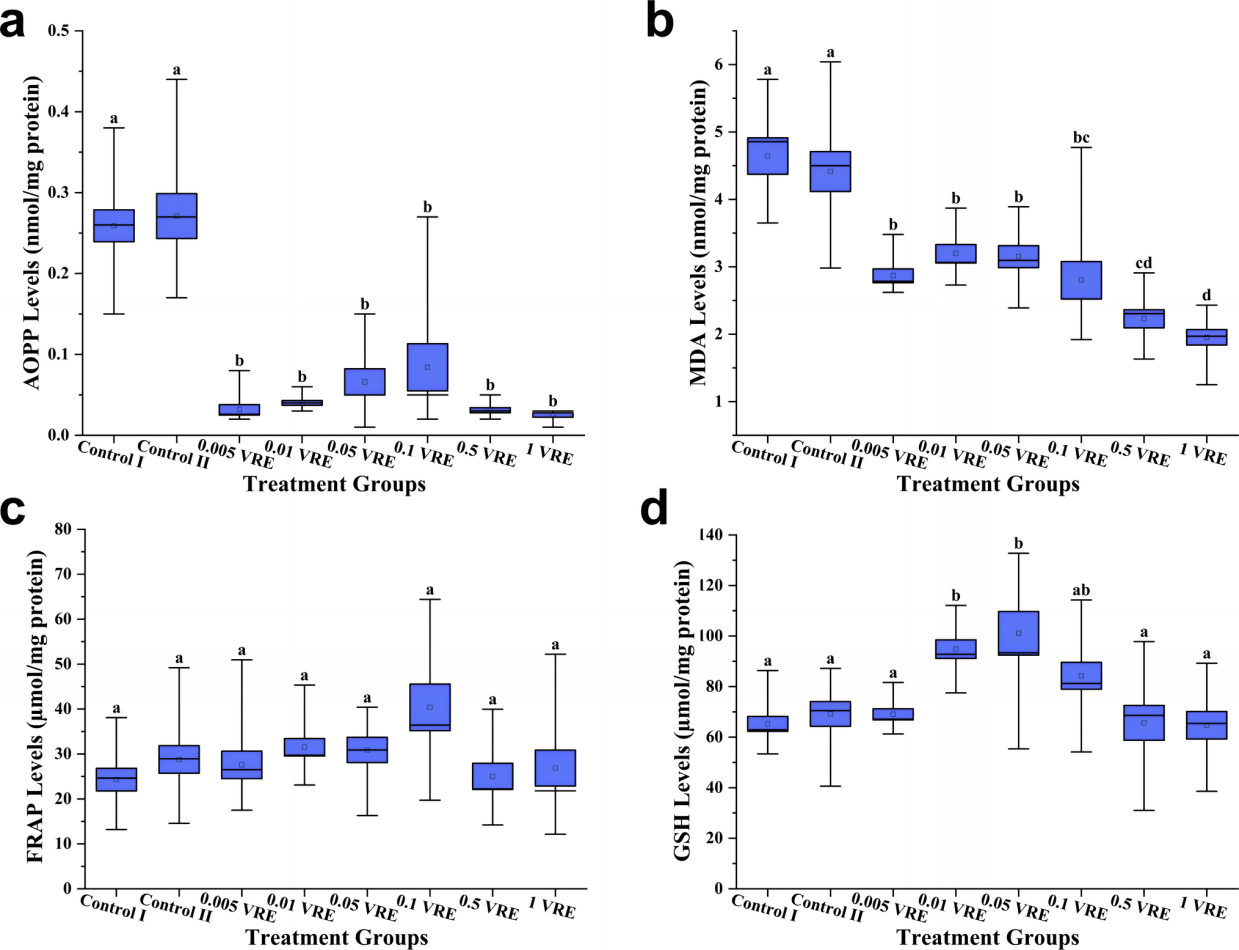
Mean ± standard error of **a** advanced oxide protein product (AOPP) and **b** malondialdehyde (MDA) contents and levels of **c** Ferric Reducing Antioxidant Power (FRAP) and **d** glutathione (GSH) on all parameters assessed in larvae of *Galleria mellonella* from the control group or those injected with different *Pimpla turionellae* venom reservoir equivalents. Boxplots represent mean + standard error; bars represent min–max values. Different letters (a–d) on the boxes indicate statistically significant differences (Dunn’s test; *p* < 0.05) between treatment groups (*n* = 10)

The effect of parasitoid venom on AOPP levels in host haemolymphs was remarkably decreased at the venom-applied doses compared to the control groups. The mean AOPP levels (mean ± standard error) of Control I, Control II, 0.005 VRE, 0.01 VRE, 0.05 VRE, 0.1 VRE, 0.5 VRE and 1 VRE groups were 0.26 ± 0.0197, 0.27 ± 0.0277, 0.03 ± 0.0059, 0.04 ± 0.0030, 0.07 ± 0.0162, 0.16 ± 0.0769, 0.03 ± 0.0031 and 0.03 ± 0.0027 nmol/mg protein, respectively (df1, df2 = 7, 72; *F* = 46.516; *p* < 0.001) (Fig. 2a).

Analysing the changes in MDA levels, a decrease in the amount of oxidised lipids was detected as the venom dose increased. Mean MDA levels were 4.64 ± 0.2698 in Control I, 4.41 ± 0.2962 in Control II, 2.87 ± 0.1024 in 0.005 VRE, 3.20 ± 0.1337 in 0.01 VRE, 3.15 ± 0.1635 in 0.05 VRE, 2.80 ± 0.2765 in 0.1 VRE, 2.23 ± 0.1340 in 0.5 VRE and 1.95 ± 0.1142 nmol/mg protein in 1 VRE (*F* = 57,029; *p* < 0.001). While MDA level was statistically at the highest level in control groups, it was determined at a high level in 0.005 VRE, 0.01 VRE and 0.05 VRE groups, at an intermediate level in 0.1 VRE group, at a low level in 0.5 VRE group and at the lowest level in 1 VRE group (Fig. 2b).

FRAP analysis of haemolymph of host larvae revealed no statistically significant effect of parasitoid venom on antioxidant power. The mean FRAP levels of Control I, Control II, 0.005 VRE, 0.01 VRE, 0.05 VRE, 0.1 VRE, 0.5 VRE and 1 VRE groups were 24.30 ± 2.5220, 28.78 ± 3.0659, 27.59 ± 3.0610, 31.51 ± 1.9267, 30.90 ± 2.8139, 40.38 ± 5.1860, 25.02 ± 2.9184 and 28.87 ± 5.5828 µmol/mg protein, respectively (*F* = 11,472; p = 0.119; Fig. 2c).

Although low and high doses of parasitoid venom did not alter GSH levels compared to the control group, moderate doses showed antioxidant effect. Mean GSH levels were 65.241 ± 2.9641 for Control I, 69.18 ± 4.8848 for Control II, 69.03 ± 2.1904 for 0.005 VRE, 94.80 ± 3.6828 for 0.01 VRE, 101.08 ± 8.6280 for 0.05 VRE, 84.28 ± 5.3120 for 0.1 VRE, 65.64 ± 6.8916 for 0.5 VRE and 64.68 ± 5.4322 µmol/mg protein for 1 VRE (*F* = 7395; *p* < 0.001). While 0.01 VRE and 0.05 VRE doses showed statistically high antioxidant effect, 0.1 VRE dose showed moderate antioxidant effect. At other doses, no change was determined in terms of statistical significance compared to the control groups (Fig. 2d). When the results of biochemical analyses were evaluated cumulatively, it was observed that parasitoid venom decreased oxidative stress and showed antioxidant effect.

**Fig. 3:**
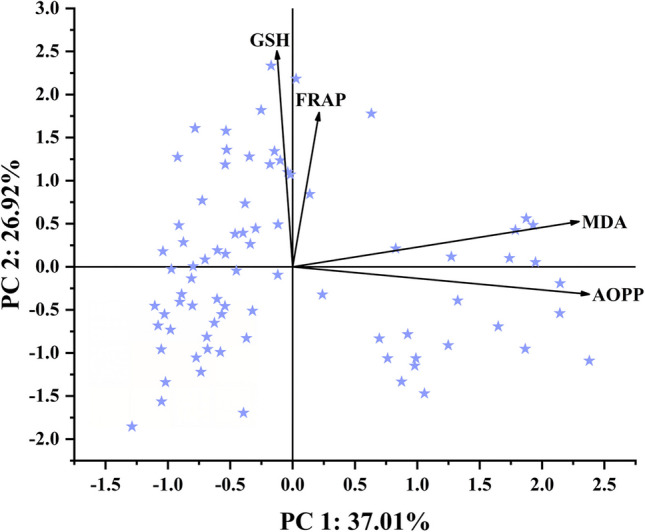
Principal component analysis biplot of levels of oxidative stress indicators (advanced oxidised protein products (AOPP) and lipid peroxidation (MDA)) and antioxidants (Ferric Reducing Antioxidant Power (FRAP) and glutathione (GSH))

According to the results of PCA, it was observed that 63.94% of the variation in oxidative parameters was explained by the first two components (PC 1 and PC 2). PC 1 explained 36.77% and PC 2 explained 27.17% of the variation (Fig. 3). MDA and AOPP were positively correlated with PC 1 with rates of 83.99% and 87.01%, respectively, while FRAP and GSH were positively correlated with PC 2 with rates of 59.45% and 83.18%, respectively. PC 1 and PC 2 described 73.58% of the variance of MDA, 76.81% of the variance of AOPP, 35.95% of the variance of FRAP and 69.41% of the variance of GSH (Table 2).

**Table 2:** Principal component analysis results of oxidative parameters

	Descriptives	Component loadings with varimax rotation**		Communalities
	Mean ± SD*	Component 1	Component 2	Extraction
**MDA**	3.1564 ± 1.0801	**0.8399**	0.1744	0.7358
**AOPP**	0.1100 ± 0.1346	**0.8701**	− 0.1054	0.7681
**FRAP**	29.6676 ± 11.8319	0.0779	**0.5945**	0.3595
**GSH**	76.7406 ± 21.2540	− 0.0462	**0.8318**	0.6941

*SD, standard deviation

**Large loadings (> 0.4) are highlighted in boldface

### Genotoxic Effects of Venom

**Fig. 4:**
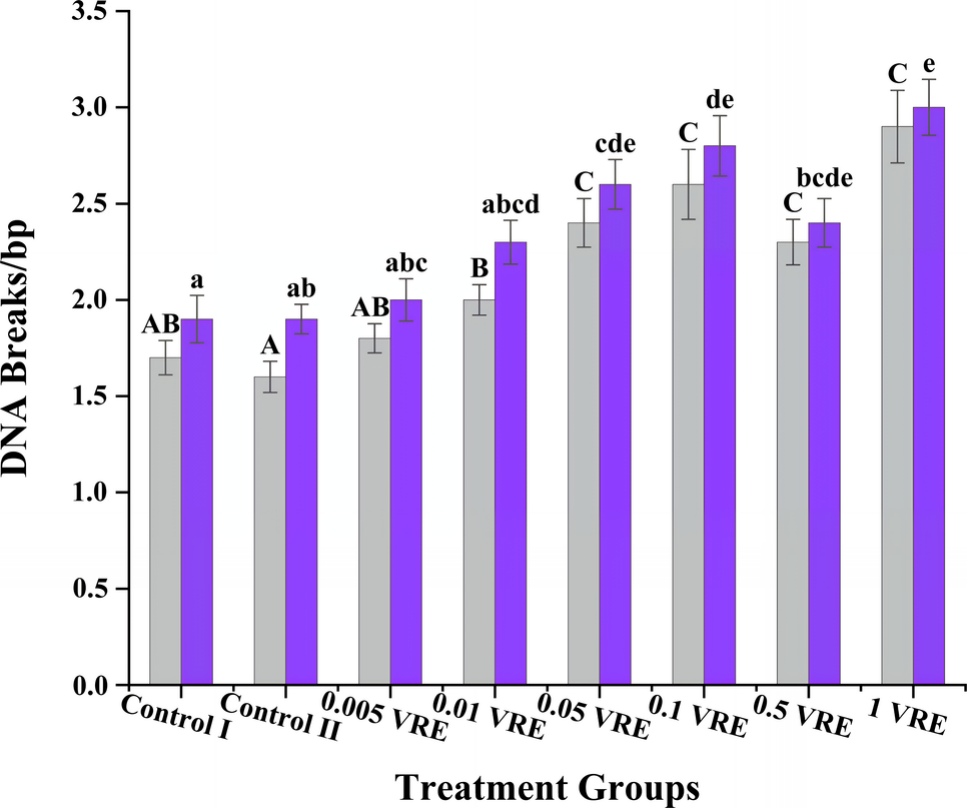
Genotoxic evaluation of different venom doses of *Pimpla turionellae* based on double strand breakage (DSB) rates under neutral conditions and double and single strand breakage (DSB + SSB) rates under alkaline conditions. Different letters on the columns indicate statistically significant differences (Tukey’s HSD; *p* < 0.05) between groups (*n* = 10). Uppercase letters (A–C) indicate the alteration of DNA damages in neutral conditions (grey boxplots), and lowercase letters (a–e) indicate the alteration of DNA damages in alkaline conditions (purple boxplots). Values are determined as mean ± standard error

Following the EAsy-GeL method, it was detected that the parasitoid venom caused damage in host DNA in parallel with the dose. Under neutral conditions, the average DSB rates per 1 bp in Control I, Control II, 0.005 VRE, 0.01 VRE, 0.05 VRE, 0.1 VRE, 0.5 VRE and 1 VRE groups were calculated as 1.7 ± 0.0894, 1.6 ± 0.0809, 1.8 ± 0.0757, 2 ± 0.0790, 2.4 ± 0.1263, 2.6 ± 0.1814, 2.3 ± 0.1183 and 2.9 ± 0.1887, respectively (*F* = 50.257; *p* < 0.001), while the mean DSB + SSB ratios per 1 bp in alkaline conditions were 1.9 ± 0.1232, 1.9 ± 0.0767, 2 ± 0.1097, 2.3 ± 0.1137, 2.6 ± 0.1291, 2.8 ± 0.1565, 2.4 ± 0.1263 and 3 ± 0.1455 (*F* = 10.466; *p* < 0.001). Considering the differences between the values in statistical significance, low level DNA breakage rates were noted in the control groups and in the 0.005 VRE group, the lowest venom dose group, while an average level of DNA breakage rate was observed in the 0.01 VRE group and high level DNA breakage rates were detected at higher doses (Fig. 4).

### Effects of Venom on Global DNA Methylation

**Fig. 5:**
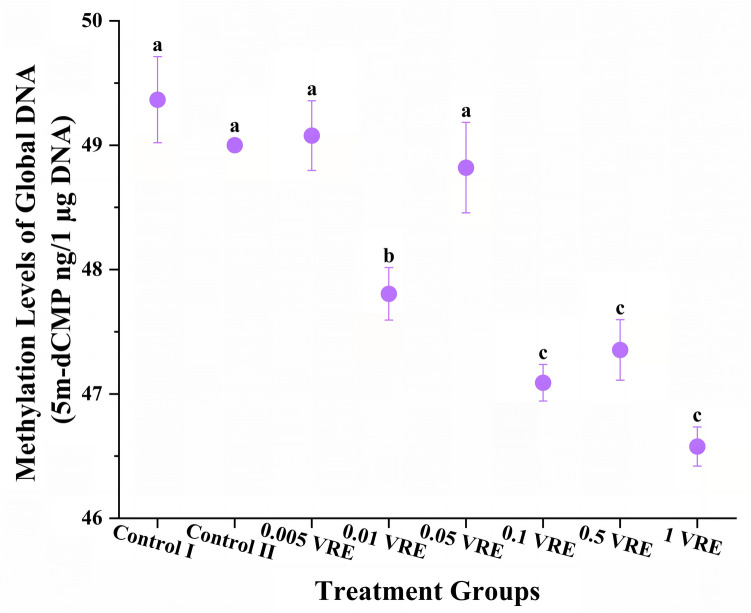
DNA methylation levels of global DNA of *Galleria mellonella* treated with different doses of *Pimpla turionellae* venom based on 5-methyldeoxycytidine monophosphate (5 m-dCMP) peak areas determined by high-performance liquid chromatography. Different letters (a–c) in the plot indicate statistically significant differences (Tukey’s HSD; *p* < 0.05) between groups (*n* = 10). Values are determined as mean ± standard error

Examination of the effect of parasitoid venom on methylation levels in host DNA pointed out that it caused demethylation. The mean 5 m-dCMP levels of Control I, Control II, 0.005 VRE, 0.01 VRE, 0.05 VRE, 0.1 VRE, 0.5 VRE and 1 VRE groups were 49.25 ± 0.3314, 49.00 ± 0.0105, 49.11 ± 0.2522, 47.92 ± 0.2206, 48.80 ± 0.3260, 47.03 ± 0.1434, 47.30 ± 0.2237 and 46.69 ± 0.1834 respectively (*F* = 53.94; *p* < 0.001). DNA methylation rates of 0.005 VRE and 0.05 VRE groups did not show statistically significant differences compared to the control groups. While DNA methylation was determined at a moderate level in the 0.01 VRE group, the DNA methylation rate was found to be the lowest at the highest doses (Fig. 5). When evaluated collaboratively in terms of oxidative, antioxidant, genotoxic and percentage DNA methylation, it was determined that 0.01 VRE dose is the most therapeutically effective dose, due to low oxidation, high antioxidation, moderate genotoxicity and demethylation effects and thus potentially favourable effects on the immune system.

### Relationships between Effects Observed on the Host Following Injection of Parasitoid Venom

**Fig. 6:**
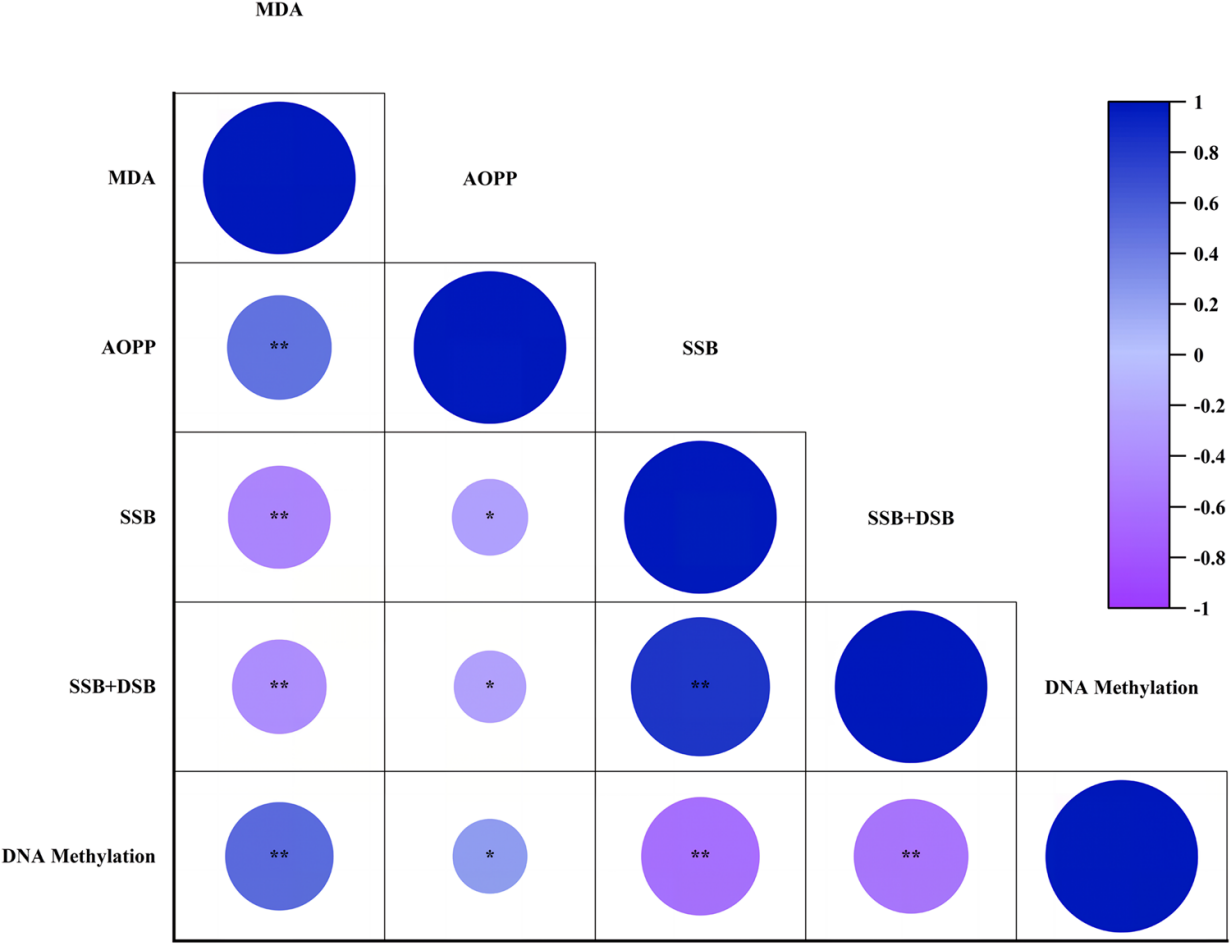
Multivariate correlation analysis plot between lipid peroxidation (MDA), advanced oxidised protein products (AOPP), double strand breaks (DSB), double and single strand breaks (DSB + SSB) and DNA methylation effects of *Pimpla turionellae* venom. * symbol indicates weak correlations between the parameters based on 2-tailed sigma value (*p* < 0.05). ** symbol indicates strong correlations between the parameters 2-tailed sigma value (*p* < 0.01)

MCA based on Pearson’s method revealed that MDA data were strongly correlated with AOPP, DSB, DSB + SSB and DNA methylation data, DSB data were strongly correlated with DSB + SSB and DNA methylation data and DSB + SSB data were strongly correlated with DNA methylation data, while AOPP data were weakly correlated with DSB, DSB + SSB and DNA methylation data. MDA was positively correlated with AOPP and DNA methylation with 47.0% and 50.5%, respectively, and negatively correlated with DSB and DSB + SSB with 45.3% and 38.2%, respectively. AOPP showed 25.1% and 22.5% negative correlation with DSB and DSB + SSB, respectively, and 24.0% positive correlation with DNA methylation. It was calculated that the alteration between genotoxic parameters resulted in a positive correlation of 83.0%. DNA methylation data were 60.7% and 56.3% negatively correlated with DSB and DSB + SSB data, respectively (Fig. 6). The colour and size of the circles in the graph change proportionally with the correlation coefficient.

## Discussion

*Galleria* *mellonella *has become widespread as a model organism in pharmacology, toxicity, oxidative stress and epigenetic studies because they have innate immune mechanisms similar to vertebrates, have low cultivation costs, are easy to maintain, have a short life cycle, do not necessitate special laboratory equipment and do not require ethical approval in studies (Ellis et al. 2013; Harding et al. 2013; Kwadha et al. 2017; Ménard et al. 2021; Mukherjee et al. 2017; Ramarao et al. 2012; Singkum et al. 2019; Tsai et al. 2016; Wojda et al. 2020). However, although *G*. *mellonella* has immune memory against reinfections through epigenetic inheritance, it does not have antigen-specific adaptive immunity as in mammals (Gallorini et al. 2024; Netea et al. 2020; Vilcinskas 2021). Furthermore, in addition to the lack of adaptive immunity, *G*. *mellonella* does not have the complex organ systems seen in mammals (Gallorini et al. 2024; Kling 2020), so the translation of the findings to human health studies may require testing in mammalian models or in vitro in human cell lines to verify the hypotheses. Intensive studies on venoms have led to the enlightenment of the immunotherapeutic potential of venom components (De Graaf et al. 2010). However, the effects of solitary parasitoid wasps on humans and mammals have not been evaluated under laboratory conditions (Moreau and Asgari 2015). Within this scope, the pharmacological effects of the venom of *P*. *turionellae*, a solitary endoparasitoid wasp species, were evaluated on *G*. *mellonella*, an alternative model organism regarding human immunity, and the therapeutic dose was determined.

Parasitoid venom consists of many complex components with a variety of functions (De Graaf et al. 2010). By infrared spectroscopy, it was revealed that *P*. *turionellae* venom has acidic nature, does not contain carbohydrates, contains phosphoric compounds and is peptide-rich (Uçkan and Gülel 1990; Uçkan et al. 2004). As a result of SDS-PAGE analyses, these peptides were determined to be intensely between 20 and 106 kDa molecular weight (Uçkan et al. 2004). Thin-layer chromatography (TLC), HPLC and proteo-transcriptomic studies revealed that the venom contains apamin, carboxylesterase, glycoside hydrolase family 1, histamine, kunitz-type protease inhibitor, mellitin, metallopeptidase M12B, noradrenaline, pacifastin-like protease inhibitor, peptide I, peptide II, phospholipase B, pimplin2, pimplin3, pimplin4, S1 A superfamily trypsin domain, serotonin, venom acid phosphatase and many other proteins (Ergin et al. 2007; Özbek et al. 2019; Uçkan et al. 2004, 2006). Hitherto, many components have been discovered in parasitoid venoms that have numerous functions such as enhancing the toxic, allergenic, inflammatory and tissue damaging effects of the venom, increasing the efficacy of venom toxins by altering their structure and conformation and protecting the toxins, paralysing the host with neurotoxic effects, suppressing immunity by haemocyte inactivation and melanisation inhibition, disrupting the developmental and reproductive activities of the host with parasitic factors for the proper maturation of parasitoid eggs, enhancing polydnaviruses that enable the eggs to gain resistance against the host immune response, providing chemical communication, supplying antimicrobial activity against bacteria and yeasts, and showing anti-inflammatory, genotoxic and anticoagulant effects (Abd El-Wahed et al. 2021; Asgari et al. 2003a; Baracchi et al. 2012; Beckage and Gelman 2004; Chen et al. 2018; Colinet et al. 2009; Czaikoski et al. 2010; Dani and Richards 2009; Danneels et al. 2015; de Souza et al. 2019; Falabella et al. 2007; Hoshina et al. 2013; Jalaei et al. 2014; Kaushik et al. 2014; Labrosse et al. 2005; Lin et al. 2019; Moore et al. 2006; Moreau and Asgari 2015; Mortimer et al. 2013; Özbek et al. 2019; Parkinson et al. 2002; Piek 1982; Price et al. 2009; Quintela and McCoy 1998; Richards and Dani 2008; Saba et al. 2017; Schmidt 2006; Shen et al. 2010; Wang et al. 2020, 2013; Wu et al. 2008; Zhang et al. 2004a, 2006; Zhu et al. 2011, 2010). de Souza et al. (2019) applied the shotgun proteomics technique to determine the protein content of the venom of the wasp *Polybia paulista* (Ihering) (Hymenoptera: Vespidae). As a result of the analyses, proteins such as cytochrome P450, cytochrome oxidase, NADH-ubiquinone oxidoreductase, NADH dehydrogenase and thioredoxin peroxidase, which are involved in protecting venom toxins from oxidative stress, were discovered. Also, the genome of a parasitoid wasp, *Trichogramma dendrolimi* (Matsumura) (Hymenoptera: Trichogrammatidae), was analysed (Zhang et al. 2023). Gene Ontology enrichment analyses demonstrated that venom-related genes play key roles in pathways such as antioxidant activity and oxidative stress response. Thus, the fact that parasitoid venoms have a protein group including antioxidant enzymes and that venom genes showed a statistically strong association with protection against oxidative stress may be explanatory mechanisms for the antioxidant effect observed on the host. In addition, a protein called Ae-γ-GT was discovered in the venom of *Aphidius ervi* (Haliday) (Hymenoptera: Braconidae), a solitary endoparasitoid wasp species (Falabella et al. 2007). This protein shows great sequence similarity with γ-glutamyl transpeptidase enzymes which play a key role in GSH metabolism (Falabella et al. 2007; Lieberman et al. 1995; Zhang et al. 2005). The increase in host GSH levels in the present study may be explained by the potential presence of such proteins in the parasitoid venom.

Çim and Altuntaş (2021) used crude venom extracts diluted with insect saline buffer (ISB) and 4 different lethal concentrations determined to observe the oxidative effects of *P*. *turionellae* venom on *G*. *mellonella* pupae (LC_10_ = 0 µg venom/2 µL ISB, LC_30_ = 0.25 µg/2 µL ISB and LC_50_ = 1 µg/2 µL ISB); the change in MDA levels was measured spectrophotometrically 1, 2 and 4 h after venom administration. As a result of the analyses, it was determined that oxidative stress declined considering the decrease in MDA levels. This conclusion is consistent with the results obtained here. On the other hand, separate and co-parasitism with the ectoparasitoid wasp *Bracon hebetor* (Say) (Hymenoptera: Braconidae) and the entomopathogenic nematode *Steinernema carpocapsae* (Weiser) (Rhabditida: Steinernematidae) increased the MDA content of host *G*. *mellonella* haemolymph (Aamer et al. 2024). Furthermore, MDA levels in haemolymph of *G*. *mellonella* larvae inoculated by gavage technique with okadaic acid, a toxin produced by dinoflagellate species, were also increased (Coates et al. 2019). This suggests that the relationship between the host immune response and the toxins exposed has an extremely important effect on the oxidative stress effect. This suggests that *P*. *turionellae* venom appears to be the most potential pharmaceutical candidate compared to other toxins in the literature when evaluated in aspect of oxidative effect.

Parasitic factors such as venom, teratocytes, polydnaviruses and virus-like particles in parasitoids are known to suppress host immunity (Asgari et al. 2003b; Beck et al. 2000; Beckage and Gelman 2004; Edson et al. 1981; Ferrarese et al. 2009; Huw Davies et al. 1987; Kroemer and Webb 2004; Makwana et al. 2023; Schmidt 2006; Schmidt et al. 2001; Strand and Noda 1991; Zhang et al. 2004b; Zhu et al. 2011). Human renal mesangial cell (HRMC) lines were treated with 0, 0.0078125, 0.015625 and 0.0625 VRE venom doses of the ectoparasitoid *Nasonia vitripennis* (Walker) (Hymenoptera: Pteromalidae) and it was observed that the expression of genes related to metabolic pathways changed significantly 4 h after venom treatment (Siebert et al. 2019). MT-RNR2-like 1 gene, which shows the highest alteration in gene expression due to the dose of venom and down-regulated significantly, shows sequence homology with Humanin 1 gene, which is effective in superoxide dismutase (SOD)–mediated antioxidant defence (Siebert et al. 2019; Sreekumar et al. 2016; Zhao et al. 2012). This down-regulation suggests that the venom has a suppressive effect on the antioxidant system. Besides, it was observed that *P*. *turionellae* venom decreased the activities of SOD, glutathione-s-transferase (GST) and catalase (CAT) antioxidant enzymes on host *G*. *mellonella* 1, 2 and 4 h after venom injection and parasitising treatments (Çim and Altuntaş 2021). However, in the current study, GSH levels increased at low doses 24 h following venom administration. Meanwhile, the presence of iron in the structure of haemoglobin, the protein carrying oxygen in blood, and copper in the structure of haemocyanin, the protein carrying oxygen in haemolymph, explains the lack of difference in FRAP levels. Because the insufficient amount of iron in haemolymph could not reflect the amount of change sufficiently. Here, in contrary to the literature, the increase in antioxidant levels suggests that although the venom suppresses immunity in the short term, it improves immunity in the long term. Confirming this prediction, MDA and hydrogen peroxide levels were examined to observe oxidative stress effects in host *Bombyx mori* (L.) (Lepidoptera: Bombycidae) haemocytes 0, 2, 6, 12, 24, 48 and 72 h after parasitism by the endoparasitoid *Exorista bombycis* (Meigen) (Diptera: Tachinidae) (Makwana et al. 2017). The parasitoid venom increased the MDA level in the first 24 h with a maximum level at 12 h, but 48 and 72 h after parasitism, the MDA level was even lower than the MDA level of non-parasitised hosts. Hydrogen peroxide level increased in the first 48 h, but decreased in the 72nd hour. In addition, host *Chloridea virescens* (Fabricius) (Lepidoptera: Noctuidae) haemocytes were exposed to ovarian proteins and fractions characterised from *Toxoneuron nigriceps* (Viereck) (Hymenoptera: Braconidae) venom for 30 min and 1 and 2 h (Salvia et al. 2022, 2021). Staining of haemocytes with 2,7 dichlorodihydrofluorescein acetate under microscope revealed that ovarian proteins induced oxidative stress. According to these information, it is noteworthy that the dose of venom, the exposure period to venom and the time elapsed after exposure to venom are the determining factors for oxidative stress induction.

Due to the DNA damage restoring effects of *Apis mellifera lamarckii* (Cockerell) (Hymenoptera: Apidae) and *A*. *mellifera* (L.) venoms on propionic acid–induced (Khalil et al. 2015) and 915 MHz microwave radiation–induced (Gajski and Garaj-Vrhovac 2009) DNA damage, respectively, it was believed that there are components in Hymenoptera venom that can reduce environmental genotoxicity (Sjakste and Gajski 2023). However, the venoms of *A*. *mellifera* (Hoshina and Marin-Morales 2014) and *P*. *paulista* (Hoshina et al. 2013) did not show any restoring effect on methanesulfonate-induced genotoxicity in human hepatoblastoma (HepG2) cell line. Moreover, pharmaceutically bioactive components found in Hymenopteran venom, such as phospholipases, hyaluronidases and mastoparan (Luo et al. 2022) and peptides such as melectin (Liang et al. 2021), endonuclease-like venom protein of *Pteromalus puparum* (L.) (Hymenoptera: Pteromalidae) (PpENVP) (Wang et al. 2021) and mellitin (Gajski and Garaj-Vrhovac 2008) increase the genotoxic impacts of parasitoid venom (Sjakste and Gajski 2023). Based on the parameters of tail density, tail length, tail migration and tail moment as a result of alkaline comet analysis, it was noted that *P*. *turionellae* venom increased genotoxicity in *G*. *mellonella* pupal haemocytes depending on the dose injected and the time elapsed after injection (Çim and Altuntaş 2021). According to this study, in order to measure the genotoxic effects of *P*. *turionellae* venom, DSB and DSB + SSB ratios of DNA isolated from host *G*. *mellonella* larvae were determined using the EAsy-GeL method, which is an alternative to the Comet assay. The result of the analysis showed that, similar to Comet data (Çim and Altuntaş 2021), *P*. *turionellae* parasitoid venom increased DNA damage proportionally with increasing dose. A significant increase in the genotoxicity of *G*. *mellonella* larval haemocytes separately and co-parasitised with *B*. *hebetor* and *S*. *carpocapsae* was observed compared to unparasitised ones (Aamer et al. 2024). In another study, different doses of *P*. *paulista* venom (10 μg/mL, 5 μg/mL, 1 μg/mL, 500 ng/mL, 100 ng/mL, 50 ng/mL, 10 ng/mL, 1 ng/mL, 100 pg/mL and 10 pg/mL) were applied to HepG2 cell line (Hoshina et al. 2013). High doses of the venom (10 μg/mL, 5 μg/mL and 1 μg/mL) showed genotoxic impacts. On the basis of this knowledge repository, it is concluded that parasitoid venom shows genotoxic effects in parallel with the dose of venom due to the components in its content.

At 4, 8 and 24 h after parasitisation by *P*. *turionellae*, 5 m-dCMP levels of DNA isolated from *G*. *mellonella* pupae were measured by HPLC (Özbek et al. 2020). Based on these measurements, it was determined that DNA methylation levels decreased 4 and 8 h after parasitism compared to the control. In addition, the expression levels of DNA methyltransferase 1-associated protein and DNA cytosine 5-methyltransferase genes, which are involved in DNA methylation processes, down-regulated according to qPCR data. Our findings show that there is a negative correlation between venom dose and host global DNA methylation level. Consequently, exposure to high parasitoid venom dose lowers DNA methylation levels in a short period of time. Epigenetic mechanisms such as DNA methylation play a role in the susceptibility and resistance mechanisms of hosts against pathogens and parasites (Mukherjee and Dobrindt 2022; Sommer 2020; Vilcinskas 2016; Zhang and Cao 2019). When considered as a whole, the activation of host immune genes after venom exposure explains the decline in DNA methylation levels. Gene Ontology enrichment analyses were performed on proteins expressed by *Periplaneta americana* (L.) (Blattodea: Blattidae) parasitised by the solitary endoparasitoid jewel wasp *Ampulex compressa* (Fabricius) (Hymenoptera: Ampulicidae) 10 min and 24 h after parasitism (Kaiser et al. 2019). Proteins 10 min after parasitism are associated with histone and protein methylation processes. This suggests that *P*. *turionellae* venom functions as a DNA demethylation agent, whereas *A*. *compressa* venom may be a potential methylation agent. It is crucial to increase the number of studies examining the effects of different parasitoid venoms on the global DNA methylation levels of hosts and the expression levels of genes related to DNA methylation pathways in order to expand the literature in this field and to identify effective demethylation agents.

In an effort to demonstrate the pharmacological value of *P*. *turionellae*, different doses of venom sac contents were applied to *G*. *mellonella*. Comparison of the data obtained here and the studies in the literature regarding oxidative stress, genotoxicity and DNA methylation revealed that the pharmaceutical properties of *P*. *turionellae* venom were higher than other parasitoid venoms. PCA data revealed that there were two components (PC 1 and PC 2) explaining the variances in oxidative parameters. Therefore, PC 1 is the factor that gathers oxidative stress markers under a common framework, while PC 2 defines the constituents in the antioxidant system. In addition, antioxidant, genotoxic and demethylation effects correlated with each other. High venom doses reduced oxidative stress and methylation according to biochemical and chromatographic measurements. However, electrophoretic analyses showed that these doses resulted in very high DNA damage. Because the potential presence of enzymes in the venom content, such as oxidative stress protective proteins and glutamyl transpeptidase, which trigger GSH production, induced an antioxidant effect on the host. In addition, proteins found in *P*. *turionellae* venom, such as mellitin, increase genotoxicity. This may be due to the activation of host immune genes by lowering methylation levels to ameliorate the damage caused by genotoxicity. Therefore, it was determined that 0.01 VRE dose may be the ideal immunotherapeutic dose for *P*. *turionellae* in the context of low level AOPP, high level antioxidant, moderate level MDA and genotoxicity and demethylation effects. In order to identify alternative pharmaceutical candidates among parasitoid venoms, oxidative/toxic effects, allergy/pain-inducing potentials, molecular mechanisms of action of different parasitoids can be determined and their venom contents can be elucidated by multi-omics approaches. Also, possible lethal and therapeutic doses for humans can be determined by studying parasitoid venoms in vitro in human cell lines. Furthermore, pharmacological findings can be obtained for human health by testing the potential effects forecasted from model organisms.

## Data Availability

The datasets used and/or analysed during the current study are available from the corresponding author on reasonable request.
